# Efferocytosis in myocardial infarction: the regulatory core from inflammation resolution to cardiac repair

**DOI:** 10.3389/fimmu.2026.1782933

**Published:** 2026-03-09

**Authors:** Chao Meng, Shiyi Tao, Yonghao Li, Jun Li, Xuanchun Huang, Xiao Xia, Yiying Liu

**Affiliations:** 1 Department of Cardiology, Guang’anmen Hospital, China Academy of Chinese Medical Sciences, Beijing, China; 2 Graduate School, Beijing University of Chinese Medicine, Beijing, China

**Keywords:** cardiac repair, efferocytosis, immunometabolism, inflammation resolution, myocardial Infarction

## Abstract

Effective tissue repair after acute myocardial infarction (MI) critically depends on the timely and orderly resolution of inflammation. This review systematically elaborates the core “directorial” role of efferocytosis—the immunologically silent clearance of apoptotic cells—which orchestrates the post-MI immune microenvironment from inflammation to repair through precise spatiotemporal regulation. We dissect its complete molecular program, from “find-me/eat-me” signals and TAM-TIM receptor synergy to metabolic-transcriptional reprogramming that drives repair. A key focus is how efferocytic dysfunction (e.g., MerTK cleavage, CD47 upregulation, Lgmn blockade) triggers a self-perpetuating vicious cycle of failed clearance, sustained inflammation, and repair collapse, leading to adverse remodeling and heart failure. Critically, we highlight the context-dependent duality of key molecules, emphasizing that therapeutic success requires restoring physiological balance rather than maximal pathway activation. Building on this mechanistic understanding, we review multi-dimensional strategies—disabling “don’t-eat-me” signals, enhancing degradation capacity, and reprogramming the immune microenvironment—while critically analyzing translational challenges. Finally, we envision a paradigm shift toward spatially targeted, temporally precise interventions that actively guide repair, laying a theoretical foundation for innovative efferocytosis-directed therapies.

## 1 Introduction

Following myocardial infarction (MI), damage-associated molecular patterns (DAMPs) released by dying cardiomyocytes trigger a robust acute inflammatory response, an essential prelude to tissue repair. However, the failure of this response to resolve in a timely and orderly manner directly leads to dysregulated repair, driving the vicious progression of pathological ventricular remodeling (VR) and heart failure (HF) (1, 2). Within this complex pathological network, the “double-edged sword” nature of inflammation is strikingly evident: the precision of its temporal regulation, rather than its mere presence or absence, is the key determinant of cardiac fate (3, 4). Consequently, unraveling the intrinsic regulatory program that actively shifts inflammation from a “pro-inflammatory” to a “pro-reparative” resolution state has emerged as a central scientific question for improving post-MI outcomes (5, 6).

In recent years, revolutionary progress has been made in the study of efferocytosis—the immunologically silent clearance of apoptotic cells by phagocytes. It is no longer viewed as a passive “cleanup” process but is being redefined as the “director” of the temporal evolution of the post-MI immune microenvironment (7, 8). We emphasize, however, that efferocytosis is not the sole “director” of repair; it operates within a complex “production team,” engaging in close interplay and mutual calibration with neuroendocrine systems (e.g., the renin-angiotensin-aldosterone system), direct cytokine signaling networks, and other cell death clearance programs (e.g., autophagy). This core status is manifested at three levels. First, by clearing apoptotic cells, it cuts off the continued release of DAMPs at the source, creating a prerequisite for inflammation resolution (9, 10). Second, and more proactively, successful efferocytosis actively reprograms the immune function of phagocytes (primarily macrophages) (11, 12). For instance, a recent study by Gong et al. in Cell Death and Differentiation revealed that following efferocytosis, TREM2+ macrophages, via the SYK-SMAD4 signaling axis, downregulate the expression of the mitochondrial NAD+ transporter SLC25A53 (7). This metabolic reprogramming event leads to a “breakpoint” in the tricarboxylic acid cycle and the production of itaconate, which is secreted into the microenvironment as a key signaling molecule, inhibiting cardiomyocyte apoptosis and promoting fibroblast proliferation, thereby directly linking efferocytosis to cardiac repair.

Nevertheless, this repair “drama” directed by efferocytosis can be disrupted by the dysregulation of key steps (13). Among these, the overload of the lysosomal degradation pathway is a critical bottleneck leading to efferocytic function collapse and repair failure (14). Pioneering work by Liu et al. in Advanced Materials demonstrated that the lysosomal cysteine protease legumain (Lgmn) is essential for sustaining the continuous efferocytic capacity of macrophages (8). Post-MI, the massive accumulation of apoptotic cells can overload the phagolysosomal degradation function of macrophages. Their study, using lipid nanoparticles to deliver mRNA for the *in situ* engineering of chimeric antigen receptor macrophages (CAR-MΦ) with Lgmn, significantly increased their intracellular degradation flux, relieved the “blockage” in the efferocytic process, and restored the anti-fibrotic function of macrophages, providing proof-of-concept for therapeutic strategies targeting the terminal phase of efferocytosis. Notably, the function of Lgmn is cell- and context-specific; for example, in aortic dissection, macrophage-derived Lgmn can exacerbate vascular degeneration by regulating integrin αvβ3 (15), highlighting the paramount importance of spatiotemporally precise regulation of efferocytosis components.

Building on these insights, the targeting of efferocytosis has progressed from a conceptual premise to an emergent strategy for precise therapeutic intervention (16, 17). This encompasses approaches ranging from the application of nanotechnology to neutralize “don’t eat me” signaling brakes (18), to the systemic optimization of the reparative microenvironment via metabolic reprogramming (e.g., modulation of the TREM2-SLC25A53 axis (7)), and to the fabrication of smart scaffolds designed to regulate efferocytosis by drawing on tissue engineering principles (19). Consequently, an integrated therapeutic framework aimed at reinstating the critical temporal equilibrium between inflammation and repair is now materializing (20).

**Figure 1:**
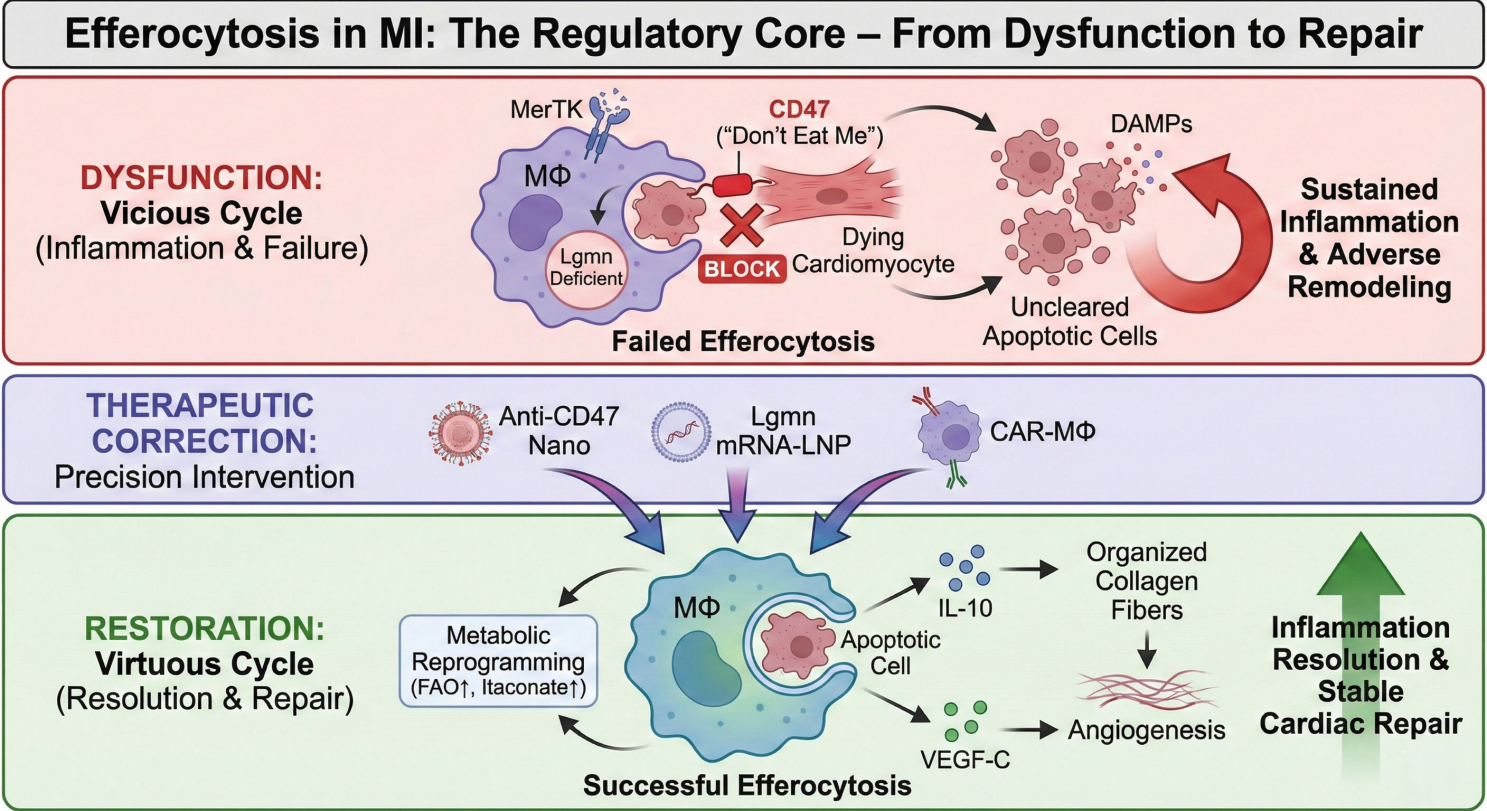
Efferocytosis as the regulatory core of post-myocardial infarction (MI) repair: From pathological dysfunction to therapeutic restoration. The schematic illustrates the pivotal role of efferocytosis in governing the transition from inflammation to cardiac repair. Top (Dysfunction): In the pathological post-MI microenvironment, a vicious cycle is established. High expression of the "don't-eat-me" signal CD47, proteolytic cleavage of MerTK (generating sMer), and Legumain (Lgmn) deficiency collectively lead to failed efferocytosis. This results in the accumulation of uncleared apoptotic cells, which undergo secondary necrosis and release DAMPs, fueling sustained inflammation ($TNF-\alpha$, $IL-1\beta$) and adverse ventricular remodeling. Middle (Therapeutic Correction): Strategic interventions target specific nodes of the efferocytic program, including Anti-CD47 nanobodies to lift phagocytic inhibition, Lgmn mRNA-LNPs to enhance lysosomal degradation, and CAR-M$\Phi$ to provide high-efficiency clearance capacity. Bottom (Restoration): Reinstating successful efferocytosis initiates a virtuous cycle. Internalization of apoptotic cells triggers metabolic reprogramming (enhanced FAO and itaconate production), which drives the secretion of reparative factors such as IL-10 and VEGF-C. These mediators promote organized collagen deposition, angiogenesis, and inflammation resolution, ultimately leading to stable cardiac repair and functional recovery.

This review will adopt the “director” perspective to systematically dissect the central role of efferocytosis in MI, focusing on (1): its dynamic evolution and regulatory network as a temporal “director” across different repair phases (2); the core mechanisms driving its functional execution, particularly recent discoveries on metabolic reprogramming and degradation pathways (3); the causal chain linking its dysfunction to adverse VR; and (4) novel strategies for temporally precise and intelligent therapies targeting efferocytosis and their clinical translation prospects. By integrating the latest cutting-edge advances, we propose a “Regulatory Core” model illustrating how the transition from a post-MI vicious cycle to a virtuous repair program is fundamentally driven by efferocytosis efficiency (**Figure 1**). Ultimately, this article aims to provide a new paradigm for understanding the programmed regulation of post-MI cardiac repair and to lay a theoretical foundation for developing innovative therapies capable of “mimicking and enhancing” the body’s own repair programs.

## 2 Cell death and the evolution of the immune microenvironment following myocardial infarction

Acute myocardial infarction (MI) initiates a sequence of spatiotemporally coordinated events encompassing cell death and immune responses, which collectively orchestrate the cardiac injury and repair microenvironment. Multiple modes of cell death are activated in accordance with the ischemic progression, culminating in the substantial release of damage-associated molecular patterns (DAMPs) that both initiate and perpetuate the inflammatory cascade. Concurrently, the infiltration and phenotypic evolution of immune cells—with the spatiotemporal efficiency of efferocytosis being particularly pivotal—directly dictate whether inflammation resolves appropriately and transitions into a high-quality reparative phase. This section will delineate the paradigm that the fate of apoptotic cells serves as the ultimate switch determining the trajectory of the immune microenvironment, a process in which efferocytosis, through its precise spatiotemporal regulation, performs the function of the “director.”

### 2.1 Cell death modalities and their role in shaping the immune microenvironment post-myocardial infarction

The cellular injury following myocardial infarction manifests as a dynamic co-existence of multiple cell death modalities, among which apoptosis plays a pivotal role in mediating the orderly resolution of inflammation and tissue repair. In contrast to necrosis, which involves rapid cell rupture and the massive release of DAMPs, apoptosis is a highly regulated form of programmed cell death that predominantly occurs in the border zone of the infarction. Apoptotic cells expose “eat-me” signals—most notably phosphatidylserine (PS) on the outer leaflet of the cell membrane—providing the critical molecular basis for subsequent recognition and clearance by macrophages, a process defined as efferocytosis (21, 22). This mechanism is essential for curbing excessive inflammation and facilitating repair.

Beyond apoptosis and necrosis, myocardial ischemia can induce other death programs. Pyroptosis, mediated by NLRP3 inflammasome activation of caspase-1, leads to Gasdermin D (GSDMD) pore formation and the release of potent inflammatory cytokines such as IL-1β and IL-18, significantly exacerbating local inflammation and amplifying myocardial ischemia-reperfusion injury (23). Ferroptosis, an iron-dependent, regulated form of cell death driven by lipid peroxidation, is also closely associated with myocardial ischemia-reperfusion injury (24). Notably, significant cross-talk exists between ferroptosis and pyroptosis pathways. Research indicates that ACSL4-dependent ferroptosis can drive the activation of the NLRP3 inflammasome, thereby triggering the pyroptosis signaling cascade. This creates a “ferroptosis-pyroptosis” amplification loop, jointly exacerbating myocardial injury and dysfunction in conditions such as pressure overload-induced heart failure (25).

These cell death modalities are pathologically interconnected. Initial ischemia causes widespread necrosis, and the released DAMPs (e.g., HMGB1, ATP) not only directly activate innate immune pathways via Toll-like receptors (TLRs) and NOD-like receptors (NLRs) (2) but can also promote apoptosis and even pyroptosis in bordering cardiomyocytes (23).

In summary, the post-MI landscape exhibits a “hierarchical burst” pattern of cell death. Vigorous death modalities like necrosis and pyroptosis act as “igniters” of inflammation, rapidly initiating and amplifying the immune response through DAMP release. In contrast, apoptosis represents a controlled, immunologically silent death that presents an opportunity for inflammation resolution. Consequently, among the various cell death events, the fate of apoptotic cells—whether they are silently cleared in a timely manner or accumulate, leading to secondary necrosis—becomes the critical “switch” determining the trajectory of inflammation. The direct orchestrator of this switch is efferocytosis. Its efficiency ultimately dictates whether the microenvironment progresses toward inflammation resolution and repair or descends into a state of persistent injury and adverse remodeling.

### 2.2 DAMP release and amplification of inflammatory signaling

DAMPs released from necrotic and secondarily necrotic cells constitute the primary driver of immune activation following myocardial infarction. DAMPs such as HMGB1, heat shock proteins, extracellular ATP, and nucleic acids, originating from ruptured cells, activate innate immune cells via pattern recognition receptors (e.g., TLR2/4, NLRP3). This triggers the activation of signaling pathways including NF-κB, leading to the robust expression of pro-inflammatory cytokines (TNF-α, IL-1β, IL-6) and chemokines (e.g., CCL2). These mediators recruit neutrophils and monocytes to the site of injury, thereby amplifying the inflammatory response (2, 26).

It is noteworthy that damaged cardiomyocytes can also actively suppress phagocytic function by inducing the proteolytic shedding of macrophage surface receptors, such as MerTK, thereby exacerbating DAMP accumulation and perpetuating inflammatory signaling (27). This exemplifies the direct consequence of failed apoptotic cell clearance: if apoptotic cells generated in the initial phase are not promptly removed, they undergo secondary necrosis, releasing additional DAMPs and establishing a self-perpetuating vicious cycle of “clearance failure → DAMP accumulation → exacerbated inflammation → further cell death” (26, 28). This process precisely illustrates how a shift in the ‘apoptotic cell fate’ switch towards ‘clearance failure’ can irreversibly propel the microenvironment toward a state of sustained inflammation. This cascade severely impedes inflammation resolution, extends the area of myocardial injury, and promotes adverse cardiac remodeling.

### 2.3 Temporal dynamics and functional switching of immune cell infiltration

**Figure 2:**
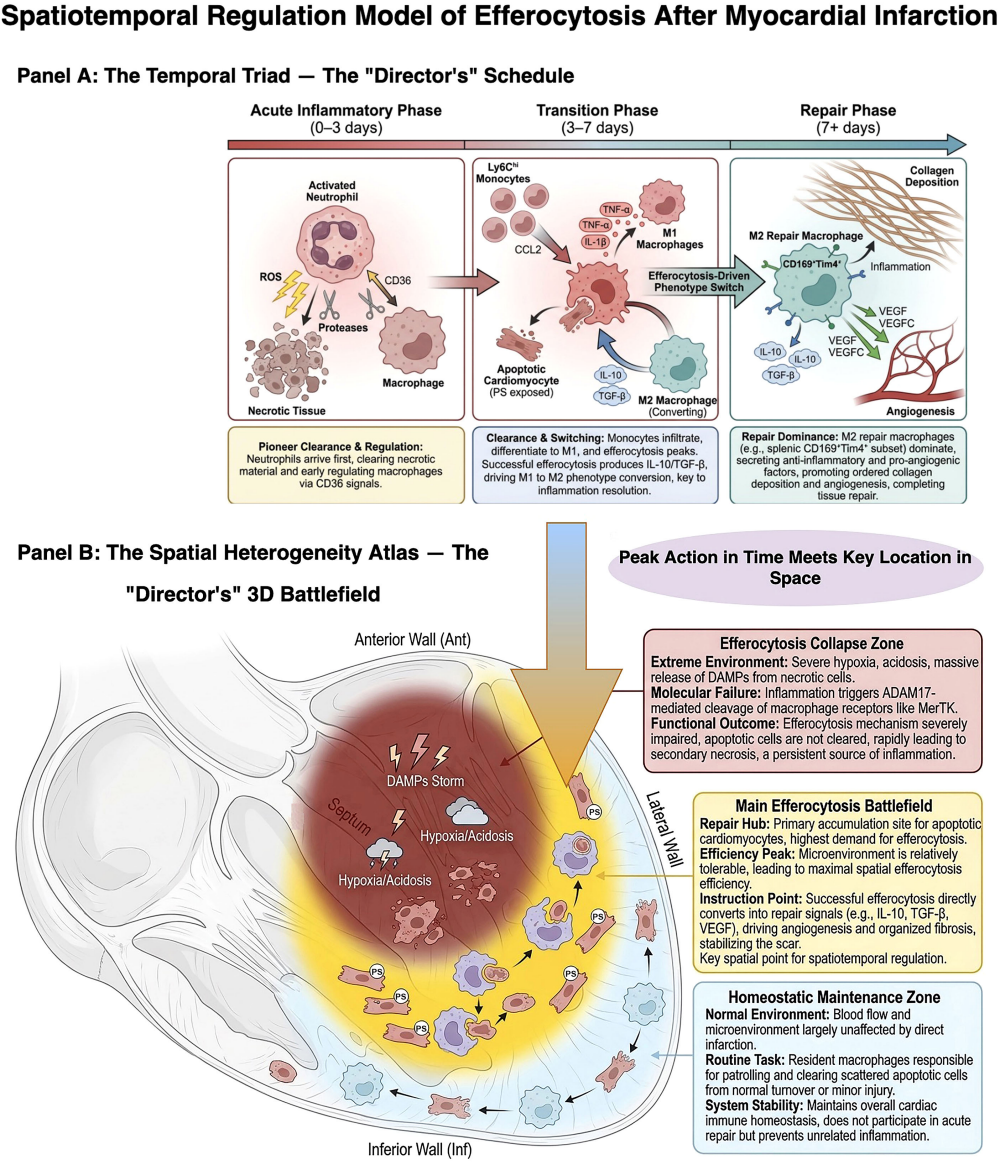
Spatiotemporal regulation model of efferocytosis after myocardial infarction.

The immune response following myocardial infarction is a highly programmed process, with its core characteristics summarized in **Figure 2**. This temporal model integrates the dynamic evolution of key cellular and molecular events, revealing that the peak of efferocytic efficiency precisely overlaps with the critical window for apoptotic cell clearance, a juncture decisive for determining the quality of cardiac repair.

During the acute inflammatory phase (days 0–3), neutrophils are the first responders to the infarcted area, clearing necrotic debris through the release of reactive oxygen species (ROS) and proteases. Emerging research further indicates that neutrophils can regulate macrophage function via molecules such as CD36, promoting their polarization towards a reparative phenotype, thereby participating in the regulation of inflammation resolution from the early stages (29, 30).

In the subsequent inflammatory peak and transition phase (days 3–7), Ly6C^hi^ monocytes are massively recruited to the heart, driven by chemokines such as CCL2, and differentiate into M1-like pro-inflammatory macrophages. These cells secrete pro-inflammatory cytokines (e.g., TNF-α, IL-1β) and matrix metalloproteinases (MMPs), simultaneously amplifying the inflammatory response and initiating extracellular matrix remodeling (31). The efferocytic activity, which peaks during this phase, is not merely a process of clearing apoptotic cells. The feedback signals generated from its successful execution—such as the upregulation of IL-10 and TGF-β—serve as a core driver for the phenotypic switch of macrophages from a pro-inflammatory M1 state to a reparative M2 state. The efficiency of this process directly determines whether inflammation can resolve smoothly.

Entering the reparative phase (beyond day 7), a critical phenotypic switch in macrophages occurs, shifting from a pro-inflammatory state towards Ly6C^lo^ and M2-like reparative phenotypes. Notably, specialized subpopulations such as splenic-derived CD169^+^Tim4^+^ macrophages are specifically recruited to the heart. By robustly expressing anti-inflammatory factors (e.g., IL-10, TGF-β) and pro-angiogenic factors (e.g., VEGF, VEGFC), they effectively suppress residual inflammation, promote orderly collagen deposition and neovascularization, thereby steering the tissue repair program (32, 33).

### 2.4 Spatial heterogeneity of efferocytosis

In addition to its precise temporal regulation, efferocytosis exhibits significant spatial heterogeneity within the infarcted heart, a feature dictated by the distinct microenvironment of each region and one that offers potential targets for precision therapy.

In the necrotic core, extreme hypoxia, acidosis, and massive necrosis create a DAMP storm. In this hostile milieu, receptors such as MerTK are prone to proteolytic cleavage by enzymes like ADAM17, severely compromising efferocytic function (27). Macrophages here are predominantly pro-inflammatory, and apoptotic cells are highly susceptible to secondary necrosis, making clearance in this zone the most formidable challenge.

The border zone, characterized by partially restored perfusion, serves as the primary site of apoptotic cell accumulation and the “main battlefield” for efferocytosis. Efferocytic efficiency is relatively highest here, orchestrated through the collaboration between cardiac tissue-resident macrophages and monocyte-derived macrophages. Successful efferocytosis in this region is most effective in inducing the secretion of anti-inflammatory factors and pro-reparative mediators like VEGF, thereby promoting angiogenesis and stabilizing the forming scar (33).

This spatial predilection for the border zone as the primary efferocytic battlefield is determined by the convergence of supply, demand, and microenvironmental feasibility (34). First, from the supply perspective, partial perfusion in this region results in the generation of predominantly clearable apoptotic cells rather than cells undergoing immediate necrosis (35). Second, from the demand perspective, apoptotic cells accumulate most abundantly in this zone, creating the peak clearance requirement (34). Third, and critically, the microenvironmental conditions here are comparatively permissive: the degree of hypoxia, acidosis, and DAMPs exposure is milder than in the necrotic core, preserving sufficient MerTK receptor function and phagocytic capacity (36). Additionally, the partially preserved vascular network provides a structural scaffold for macrophage infiltration and subsequent angiogenesis, facilitating both the delivery of phagocytes and the eventual reparative response (35).

In the remote, non-infarcted myocardium, the relatively normal microenvironment supports efferocytic activity that primarily targets scattered apoptotic cells. This process, crucial for maintaining tissue homeostasis, is mainly carried out by the intrinsic pool of tissue-resident macrophages.

Importantly, the functional heterogeneity across these zones is underpinned by the distinct origins and properties of the two major macrophage populations involved in cardiac repair. Cardiac tissue-resident macrophages, primarily of embryonic origin, exhibit rapid response kinetics and constitutively high expression of Tim4, enabling efficient “patrolling” and clearance of scattered apoptotic cells under homeostatic conditions (37–39). They are also characterized by their reliance on specific degradation enzymes such as Legumain (Lgmn) for efficient phagolysosomal processing (22). In contrast, monocyte-derived macrophages, recruited from the circulation in response to injury, possess high proliferative capacity and remarkable phenotypic plasticity (40). They are responsible for the massive clearance burden during the inflammatory peak and serve as the primary effectors of the M1-to-M2 phenotypic switch, secreting abundant reparative factors (41). However, their phenotype is more susceptible to being “locked” into a dysfunctional state by the extreme microenvironment (42). Thus, while resident macrophages safeguard baseline homeostasis and may provide early repair signals (e.g., via the TREM2 axis) (43, 44), monocyte-derived macrophages execute the large-scale repair program, orchestrating the transition from inflammation to fibrosis. This fundamental distinction underscores the need for spatially and temporally targeted therapeutic strategies that appropriately engage each population (75, 79).This spatial heterogeneity underscores the need for future therapeutic strategies to possess region-specific targeting capabilities. For instance, interventions for the necrotic core must first counteract the extreme environment and potentially supplement functional macrophages. In contrast, strategies for the border zone should focus on protecting and enhancing the efficiency of the endogenous efferocytic machinery to prevent a collapse in the repair process.

### 2.5 The central role of efferocytosis in the evolution of the immune microenvironment

Efferocytosis occupies a central position in the post-myocardial infarction (MI) immune microenvironment, serving as the critical pivot that determines whether inflammation resolves in a timely manner and transitions towards high-quality repair. Its functional state directly governs the trajectory and outcome of the immune response, perfectly exemplifying its role as the “director.”

High-efficiency efferocytosis fundamentally curtails the sustained release of DAMPs by promptly clearing apoptotic cells, thereby preventing infinite amplification of inflammatory signaling. More importantly, this process actively induces functional reprogramming of macrophages. On one hand, phagocytic signals initiated via receptors such as MerTK and CD36 (21, 28) synergize with the activation of intracellular pathways like Smad3 and STAT3 (45, 46), promoting macrophage polarization towards a reparative phenotype. On the other hand, it drives the secretion of anti-inflammatory cytokines (e.g., IL-10, TGF-β) and pro-angiogenic factors (such as VEGFC), which directly promote neovascularization and orderly fibrosis while suppressing excessive inflammation, thereby coordinating the tissue repair program. Emerging evidence also suggests that novel mediators like exosomes may be involved in fine-tuning the microenvironmental communication during efferocytosis (47).

Conversely, efferocytic dysfunction—for instance, due to proteolytic cleavage of MerTK—leads to failed apoptotic cell clearance and increased secondary necrosis. This perpetuates the pro-inflammatory environment, not only hindering macrophage polarization towards an M2 phenotype but also exacerbating myocardial injury and extracellular matrix dysregulation, ultimately accelerating adverse ventricular remodeling and the progression towards heart failure (26, 28).

It must be recognized, however, that successful repair is the product of multiple cooperating programs. For instance, direct signaling by anti-inflammatory cytokines such as IL-10 can drive M2 polarization independently of efferocytosis; conversely, sustained activation of angiotensin II constitutes a potent, pro-fibrotic parallel “instruction.” The “directorial” efficacy of efferocytosis lies, in part, in its capacity to appropriately suppress or integrate these competing signals, rather than entirely supplanting them.

Consequently, targeting efferocytosis to enhance apoptotic cell clearance efficiency or modulate associated signaling pathways for optimizing the immune microenvironment has emerged as a promising therapeutic strategy for promoting post-MI inflammation resolution and tissue repair. This fully underscores its pivotal status as the “regulatory core from inflammation resolution to cardiac repair.”

This temporal model integrates key post-myocardial infarction (MI) events. Acute Inflammatory Phase (Days 0–3): Necrosis/pyroptosis and neutrophil infiltration dominate, initiating the inflammatory cascade. Inflammatory Peak and Transition Phase (Days 3–7): Apoptosis reaches its zenith, accompanied by M1-like macrophage infiltration. Efferocytic efficiency peaks during this stage, representing the critical window for apoptotic cell clearance and determining the trajectory of inflammation. Reparative Phase (Beyond Day 7): M2-like reparative macrophages predominate, driving tissue repair. It is crucial to note that the inverted U-shaped curve of efferocytic efficiency must precisely overlap with the demand window for apoptotic cell clearance. Any factor causing a premature peak or a lag in this efficiency disrupts the temporal program, ultimately leading to suboptimal repair.

## 3 Core molecular mechanisms of efferocytosis

**Figure 3:**
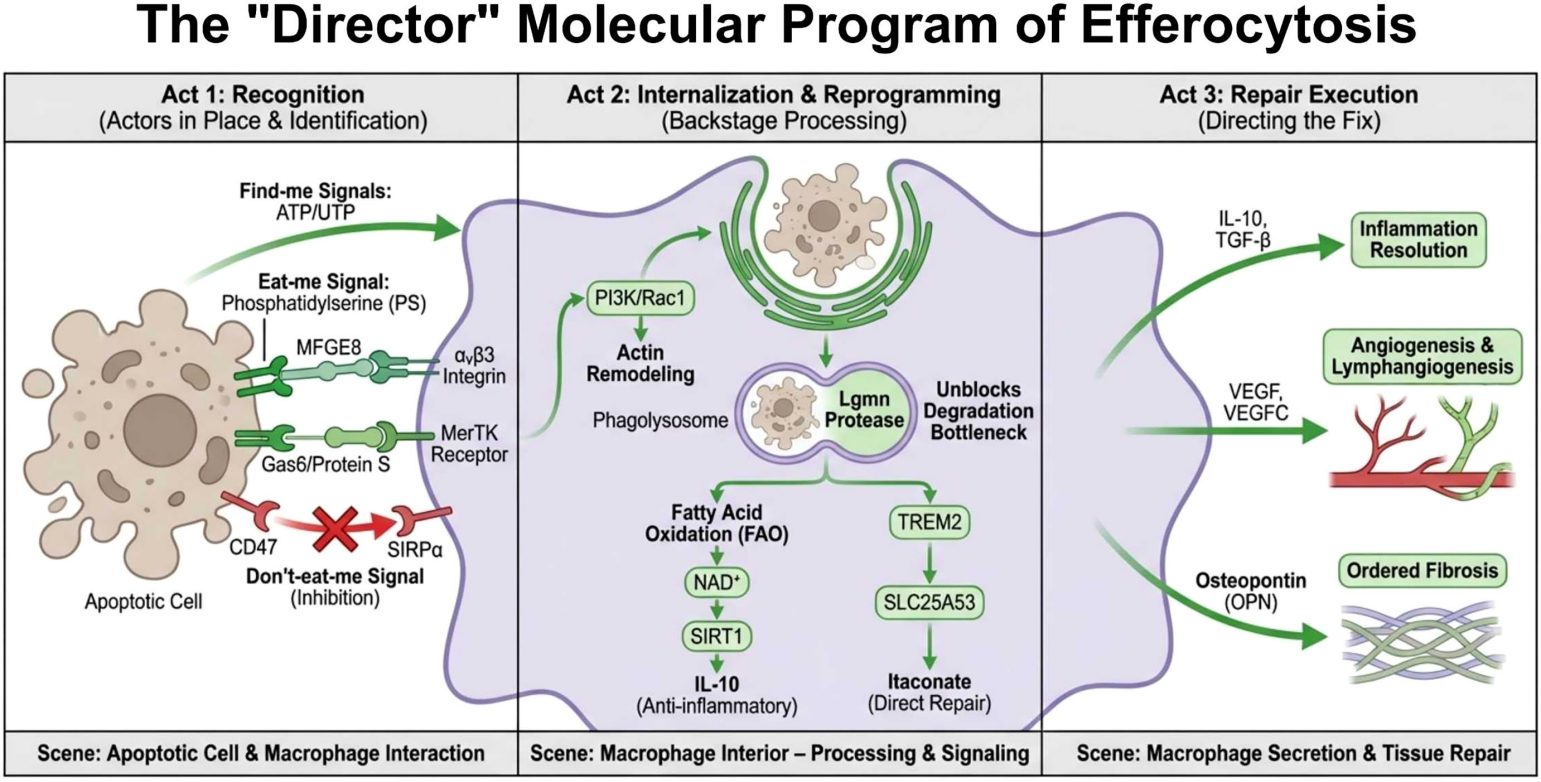
The “directorial” molecular program of efferocytosis.

The precise execution of efferocytosis following myocardial infarction relies on a coherent chain of molecular events, spanning from “recognition” to “repair.” This process is initiated by the active recruitment and molecular identification of phagocytes by apoptotic cells. It proceeds through specific receptor-ligand recognition, intracellular signal transduction, and profound metabolic-transcriptional reprogramming, ultimately culminating in the functional transition from cellular clearance to tissue repair (5). This chapter will systematically dissect this complex and sophisticated program and integrate the findings into a core mechanistic map (**Figure 3**), thereby revealing its molecular foundation as the “director” of cardiac repair.

### 3.1 “Eat-me” and “find-me” signals: directional recruitment and labeling of apoptotic cells

The initiation of efferocytosis relies on a coordinated interplay between “find-me” signals released by and “eat-me” signals exposed on the surface of apoptotic cells, which establish both a chemical gradient and a molecular tag. Early apoptotic cells actively release nucleotides such as ATP and UTP as “find-me” signals, creating a chemotactic gradient in the microenvironment. This gradient actively guides phagocytes, including macrophages, to migrate towards and accumulate at the site of injury, thereby laying the spatial groundwork for subsequent clearance (27). Concurrently, apoptotic cells execute a molecular switch on their plasma membrane: the activation of calcium-independent phospholipid scramblases coupled with the inhibition of ATP-dependent aminophospholipid translocases. This leads to the specific externalization of phosphatidylserine (PS), a phospholipid normally confined to the inner leaflet of the lipid bilayer, presenting it on the outer cell surface as the decisive “eat-me” signal for phagocyte recognition (48). This sequence completes the preparatory steps from ‘recruitment’ to ‘identification’. Although molecules such as oxidized lipids and calreticulin can serve as auxiliary “eat-me” signals, PS remains the most universal and indispensable core ligand in this process.

### 3.2 “Don’t-eat-me” signals: the physiological brake maintaining self-tolerance

To prevent the erroneous clearance of healthy cells by the phagocytic system, the body has evolved a critical physiological brake mechanism, exemplified by the CD47-SIRPα axis, known as the “don’t-eat-me” signaling system (49). The CD47 molecule is widely and highly expressed on the surface of healthy cells, serving as a marker of “self.” Upon binding to the immune receptor SIRPα on macrophages, CD47 triggers the phosphorylation of the immunoreceptor tyrosine-based inhibitory motif (ITIM) within the SIRPα intracellular domain. This recruits and activates tyrosine phosphatases such as SHP1, transducing a potent inhibitory signal. This signal effectively blocks actin cytoskeleton rearrangement and phagocytic synapse formation, thereby maintaining self-tolerance and preventing erroneous phagocytosis under physiological conditions. In the pathological context of myocardial infarction, dying cardiomyocytes aberrantly upregulate CD47 expression, thereby exacerbating the inhibition of efferocytosis—a finding that provides a rationale for its targeting in therapeutic strategies (16).

### 3.3 Bridging molecules: amplifiers of ligand-receptor connection and signal transduction

Bridging molecules play an indispensable role as “molecular glue” between “eat-me” signals and phagocytic receptors. Among them, MFGE8 recognizes PS via its N-terminal Discoidin domain and simultaneously binds to αvβ3/αvβ5 integrins on the phagocyte surface through its C-terminal RGD motif, thereby constructing a recognition bridge (50). In a parallel and complementary recognition pathway, Gas6 and Protein S bind to PS via their Gla domains and subsequently activate TAM family receptors on phagocytes through their LG domains (51). Furthermore, the expression of bridging molecules itself is subject to precise upstream regulation. For instance, the TGF-β/Smad3 signaling pathway can directly upregulate the transcriptional expression of Mfge8 in macrophages, establishing a positive feedback loop that continuously enhances efferocytic capacity (45).

### 3.4 Phagocytic recognition receptor axes: TAM-TIM coordination and the pivotal role of MerTK

The efficient internalization of apoptotic cells by phagocytes relies on the spatiotemporal and functional precision of two major receptor families: TAM and TIM. The TIM family receptors (e.g., TIM-4) are primarily responsible for the initial “tethering” and stabilization of adhesion to apoptotic cells. This is achieved through the high-affinity binding of their IgV domain to phosphatidylserine (PS) (52). In contrast, the TAM family receptors, particularly MerTK, are activated in a bridging molecule (e.g., Gas6/Protein S)-dependent manner, undertaking the core hub functions of signal amplification, phagocytic cup formation, and driving internalization (11, 53).

The coupling of these distinct functions is facilitated by their physical interaction. TIM-4, through its extracellular domain, directly binds to the IgG-like domain of MerTK. This physical connection not only anchors the apoptotic cell to the macrophage surface but, more importantly, induces a conformational change in the MerTK intracellular domain, exposing its kinase domain and thereby promoting trans-autophosphorylation. Phosphorylated MerTK subsequently provides an ideal docking site for the p85 regulatory subunit of PI3K. PI3K activation leads to the accumulation of phosphatidylinositol (3–5)-trisphosphate (PIP3) at the membrane, recruiting and activating Akt as well as downstream Rac1 guanine nucleotide exchange factors. Activated Rac1 GTPase then drives actin polymerization, forming the phagocytic cup and ultimately completing internalization. Thus, the synergistic action of TIM-4 and MerTK effectively converts the high-affinity “tethering” signal into robust “internalization” machinery, seamlessly executing the continuum from recognition to engulfment (54).

Notably, MerTK activity is dynamically regulated by proteolytic cleavage. In inflammatory environments, proteases such as ADAM17 can cleave MerTK, generating soluble sMerTK. This soluble fragment acts as a “decoy molecule,” competitively binding to its ligands, thereby creating a negative feedback inhibition on the function of membrane-bound MerTK to precisely regulate phagocytic flux (26). However, under pathological stresses like myocardial infarction, this normally precise negative feedback mechanism can become dysregulated and excessively activated. For instance, upstream stress signals like angiotensin II, via the AT1 receptor, can activate NADPH oxidase to produce reactive oxygen species, which in turn enhance ADAM17 activity through the p38 MAPK pathway (55). This leads to excessive cleavage of MerTK, establishing a vicious cycle that persistently impairs efferocytic capacity.

### 3.5 Intracellular degradation and the safeguarding of the lysosomal pathway

Following the internalization of apoptotic cells, their ultimate fate is degradation within the phagolysosome. The efficiency of this process directly determines the macrophage’s capacity for subsequent rounds of clearance. The discovery of Legumain (Lgmn) underscores the critical importance of the lysosomal degradation pathway. Lgmn is an asparaginyl endopeptidase specifically expressed by cardiac tissue-resident macrophages. Lgmn deficiency leads to significantly worsened cardiac function post-MI in mice, accompanied by the accumulation of apoptotic cardiomyocytes (15). The underlying mechanism involves a defect in intracellular calcium mobilization within resident macrophages upon Lgmn loss, resulting in reduced cytosolic calcium. When these calcium-deficient macrophages subsequently engulf apoptotic cardiomyocytes, vesicular trafficking is disrupted, and the formation of LC3-II-dependent phagolysosomes is inhibited. Mechanistically, the reduction in cytosolic calcium affects the activity of various calcium-dependent proteins. Decreased activity of calmodulin-dependent kinases may lead to insufficient phosphorylation of downstream effectors, such as Rab GTPases that regulate vesicular trafficking or proteins driving microtubule polymerization. This, in turn, hinders the directed movement, docking, and membrane fusion between phagosomes and lysosomes, ultimately resulting in failed assembly of LC3-II-labeled phagolysosomes (22). Consequently, the continuous and efficient cycle of phagocytic degradation of dead cardiomyocytes is interrupted.

Confirming its therapeutic relevance, a recent interventional study demonstrated that *in situ* engineering of chimeric antigen receptor macrophages (CAR-MΦ) via targeted lipid nanoparticle delivery of Lgmn mRNA significantly enhanced their phagolysosomal cargo degradation capacity. This approach alleviated the overload state of the efferocytic process post-MI and restored the anti-fibrotic function of macrophages (8).

### 3.6 Efferocytosis-dependent metabolic reprogramming

Successful efferocytosis represents not merely the endpoint of a physical clearance process but also the starting point for profound functional remodeling within the phagocyte itself. Following the engulfment of apoptotic cells, macrophages utilize these as critical metabolic substrates, undergoing significant immunometabolic rewiring.

Among these changes, the reprogramming of lipid metabolism is particularly prominent. The substantial uptake of fatty acids fuels mitochondrial oxidative phosphorylation via carnitine palmitoyltransferase 1a (CPT1a)-mediated fatty acid oxidation (FAO). This process not only generates ATP but, more critically, elevates the NAD^+^/NADH ratio, thereby activating the deacetylase SIRT1. SIRT1, by deacetylating histones and the transcription factor PBX1, significantly upregulates the expression of key anti-inflammatory cytokines such as interleukin-10 (IL-10), constituting a core metabolic event driving phenotypic transition (56). Concurrently, intracellular cholesterol homeostasis shifts. The phagocytic load activates the nuclear receptor Liver X Receptor (LXR), which forms a transcriptional synergy with Peroxisome Proliferator-Activated Receptor γ (PPARγ). Together, they upregulate the expression of molecules including the ATP-binding cassette transporters A1/G1 (ABCA1/ABCG1) and MerTK, while also promoting IL-10 secretion, thereby establishing a positive feedback regulatory loop that consolidates the reparative phenotype (57).

Beyond lipid metabolism, the restructuring of amino acid metabolism is equally crucial. Arginine is metabolized by arginase-1 to ornithine and subsequently to polyamines (e.g., putrescine), which activate Rac1 to enhance sustained phagocytic capacity. Meanwhile, tryptophan is metabolized by indoleamine 2,3-dioxygenase 1 (IDO1) to produce kynurenines, which activate the aryl hydrocarbon receptor (AhR) to upregulate IL-10 and TGF-β expression, collectively fostering a microenvironment conducive to inflammation resolution (11).

Notably, recent research has unveiled a novel mechanism linking efferocytosis to metabolism: the TREM2-SLC25A53-itaconate axis. Following efferocytosis, TREM2+ macrophages downregulate the expression of the mitochondrial NAD^+^ transporter SLC25A53 via the SYK-SMAD4 signaling axis. This creates a “breakpoint” in the tricarboxylic acid cycle, leading to the accumulation and subsequent secretion of itaconate. Itaconate, functioning as a crucial metabolic signaling molecule, inhibits cardiomyocyte apoptosis and promotes fibroblast proliferation, thereby directly connecting the efferocytic process to functional cardiac repair (7).

### 3.7 Transcriptional regulation and execution of the repair program

Guided by the cofactor availability and signaling molecules generated through metabolic reprogramming, the transcriptional network of macrophages is reconfigured to consolidate their reparative functions. The transforming growth factor-β (TGF-β)/Smad3 signaling axis plays a pivotal role in this process. Efferocytosis specifically activates Smad3 (but not Smad2). The activated Smad3 not only translocates to the nucleus to directly regulate the transcription of repair-related genes such as Il10 and VEGFA, but also positively feedbacks to enhance the expression of bridging molecules like Mfge8, thereby establishing a feed-forward amplification loop that augments efferocytic efficiency (45). Furthermore, a synergistic nuclear receptor network comprising PPARγ, LXR, and the retinoic acid receptor (RAR)/retinoid X receptor (RXR) integrates lipid metabolic signals to cooperatively upregulate the expression of key factors including MFGE8, IL-10, TGF-β, and VEGF, jointly promoting inflammation resolution, matrix remodeling, and angiogenesis (57).

Macrophages that have completed this dual metabolic and transcriptional reprogramming systematically execute tissue repair tasks by secreting specific effector molecules. Among these, the secretion of VEGF-C promotes lymphangiogenesis within the infarct zone, thereby accelerating the clearance of inflammatory cells and exudate and directly improving cardiac function (33). Concurrently, MerTK-positive reparative macrophages, operating within the signaling context provided by IL-10 and macrophage colony-stimulating factor (M-CSF), efficiently produce osteopontin (OPN, encoded by the Spp1 gene) in a STAT3/ERK pathway-dependent manner. OPN plays an indispensable role in mediating orderly reparative fibrosis and maintaining tissue stabilization (46).

### 3.8 Pre-programming and microenvironmental licensing of efferocytosis

The capacity of macrophages to perform high-efficiency efferocytosis is not induced solely upon contact with apoptotic cells; they can be “pre-programmed” by other cells within the microenvironment. For instance, activated N2 neutrophils can significantly upregulate the expression of MerTK, CD36, and bridging molecules MFGE8/Gas6 on macrophages via their secretome, thereby endowing them with high efferocytic flux and reparative polarity early in the inflammatory phase (40). Additionally, an intrinsic CD36→Nr4a1→MerTK signaling axis within the monocyte/macrophage lineage is crucial for the initial establishment and maintenance of a high-clearance state in macrophages (28).

### 3.9 Cell-cell communication of efferocytic signals: beyond macrophages

The influence of efferocytosis extends beyond the phagocyte itself, shaping the behavior of multiple cell types within the infarct microenvironment through direct interactions and secreted factors. This intercellular communication is critical for coordinating the multicellular repair program (58).

Crosstalk with Fibroblasts. Following successful efferocytosis, macrophages secrete a spectrum of factors that modulate fibroblast phenotype and function. Transforming growth factor-β (TGF-β) and interleukin-10 (IL-10), both upregulated during efferocytic reprogramming (45, 57), suppress the pro-inflammatory and matrix-degrading phenotype of cardiac fibroblasts while promoting their differentiation into reparative myofibroblasts, thereby facilitating orderly collagen deposition (58). Additionally, macrophage-derived extracellular vesicles enriched with microRNAs such as miR-21 have been shown to modulate macrophage polarization and promote cardiac repair following myocardial reperfusion injury, indirectly influencing fibroblast activity and extracellular matrix remodeling (59). This bidirectional communication ensures that fibroblast activity is temporally and spatially aligned with the clearance of apoptotic debris.

Crosstalk with Endothelial Cells. Beyond the well-established role of macrophage-secreted VEGF in promoting angiogenesis (33), efferocytosis-derived signals also influence endothelial function. IL-10 directly enhances endothelial barrier integrity, reducing vascular leakage and limiting inflammatory cell extravasation (60). Furthermore, extracellular vesicles released by macrophages carry pro-angiogenic cargo, including miR-126, which promotes endothelial proliferation and tube formation while suppressing adhesion molecule expression and inflammation (61). Notably, apoptotic cells themselves contribute to this intercellular network by releasing sphingosine-1-phosphate (S1P), a bioactive lipid that acts on endothelial S1P receptors to regulate endothelial barrier integrity through the balance of Gαi-Cdc42/Rac and Gα12/13-RhoA signaling pathways, thereby reinforcing the reparative microenvironment (62).

Crosstalk with Cardiomyocytes. Although the primary role of efferocytosis is to remove dying cells, the process also generates signals that impact surrounding viable cardiomyocytes. “Find-me” signals released by apoptotic cells, such as ATP and UTP, act on purinergic P2 receptors expressed on neighboring cardiomyocytes, playing a significant role in pathological left ventricular remodeling following acute myocardial infarction (63). Moreover, efferocytic macrophages secrete growth factors like insulin-like growth factor-1 (IGF-1) and hepatocyte growth factor (HGF), which have been shown to stimulate endogenous repair and regenerative mechanisms in the damaged heart, protecting stressed cardiomyocytes from apoptosis and promoting their metabolic adaptation to hypoxic conditions (64).

Collectively, this multifaceted intercellular communication network ensures that the benefits of efferocytosis propagate throughout the injured tissue, synchronizing the activities of fibroblasts, endothelial cells, and cardiomyocytes to achieve coordinated and effective cardiac repair.

## 4 Dysregulation of efferocytosis in myocardial infarction and its consequences

**Figure 4:**
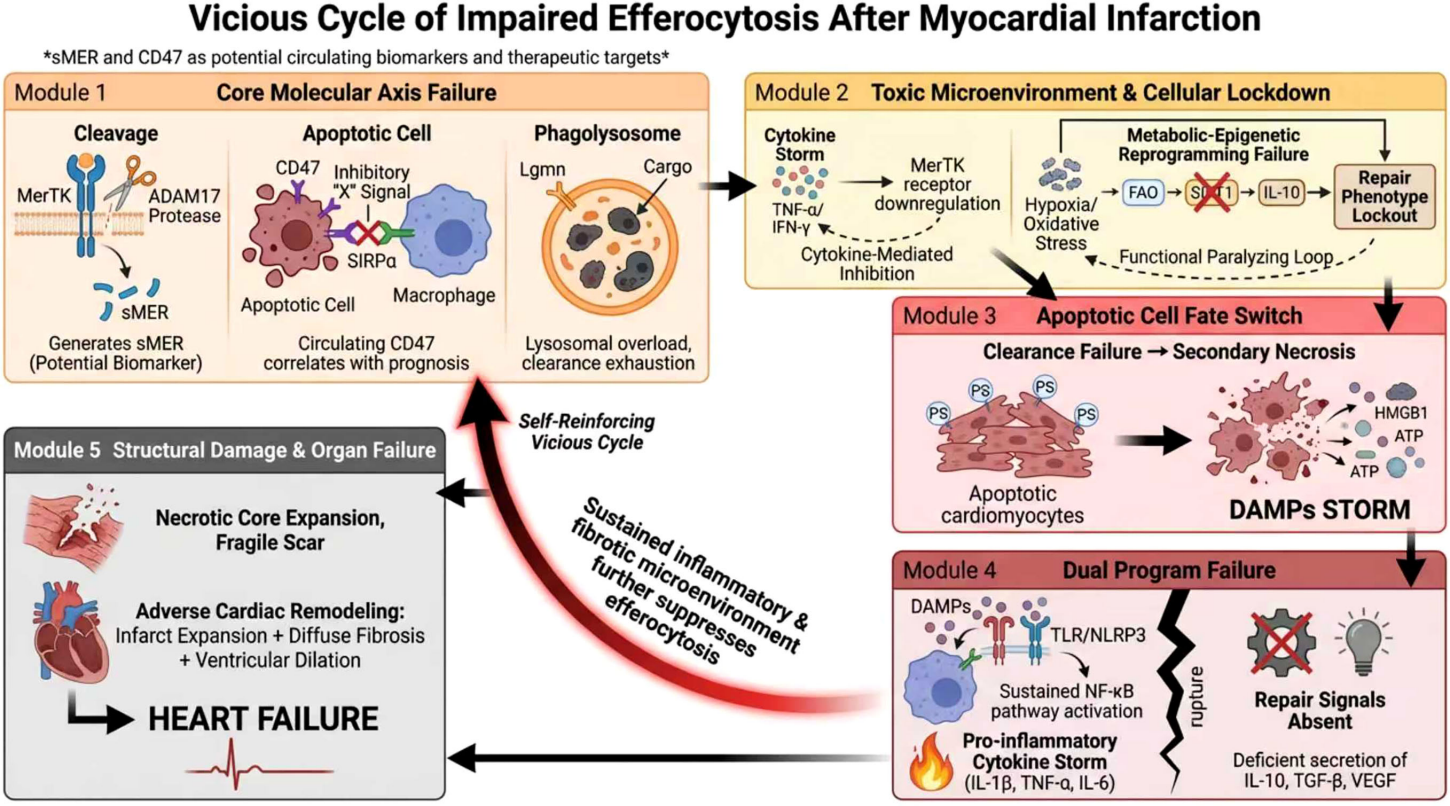
Vicious cycle of impaired efferocytosis after myocardial infarction.

Following myocardial infarction (MI), the timely clearance of apoptotic cardiomyocytes forms the cornerstone for inflammation resolution and tissue repair. Dysregulation of this sophisticated process, known as efferocytosis, triggers a cascade of pathological events spanning from molecular signaling failure to end-stage organ dysfunction. This chapter will provide a detailed dissection of this multilevel pathological cascade, which can be succinctly summarized by the following axis: “Multi-level Molecular Dysfunction → Upstream Programming Disruption → Apoptotic Cell Accumulation and Secondary Necrosis → DAMP-Driven Uncontrolled Inflammation and Failure of Repair Program Initiation → Expansion of the Necrotic Core and Adverse Cardiac Remodeling → End-Stage Heart Failure.” This chapter aims to reveal that these stages do not merely progress linearly but rather constitute a self-reinforcing ‘vicious cycle’: efferocytic failure perpetuates inflammation, and the resulting inflammatory milieu further impairs efferocytic function. These processes act as both cause and effect, jointly propelling the heart towards failure. The following sections will systematically elaborate each link within this comprehensive pathological axis, underscoring the central, pivotal role of efferocytosis in post-MI repair and the severe consequences of its dysregulation (**Figure 4**).

### 4.1 Failure of core efferocytotic regulatory axes

#### 4.1.1 Impairment of the MerTK receptor axis

The MerTK receptor, highly expressed on myeloid cells—particularly cardiac macrophages—serves as a central molecule that couples apoptotic cell recognition with the initiation of reparative programs. Its activation not only drives cytoskeletal rearrangement and phagocytic cup formation but also directly upregulates the expression of anti-inflammatory (e.g., IL-10) and pro-reparative (e.g., VEGF) factors, thereby polarizing macrophages towards a reparative phenotype (21).

However, in the inflammatory milieu post-MI, MerTK function is highly susceptible to inactivation. Proteolytic cleavage mediated by ADAM17 is a key mechanism for its functional impairment. The resulting soluble MerTK (sMer) acts as a “molecular decoy,” competitively binding to the ligands Gas6/Protein S and thereby blocking the effective activation of membrane-bound MerTK (26, 65). Upstream stress signals, such as angiotensin II, can enhance ADAM17 activity via the AT1 receptor through a pathway involving NADPH oxidase-derived reactive oxygen species and subsequent p38 MAPK signaling. This establishes a self-perpetuating positive feedback loop: “Ang II – ROS – p38 MAPK – ADAM17 – MerTK cleavage,” which persistently compromises efferocytic capacity (66). Furthermore, unidentified factors released by dying cardiomyocytes can activate ADAM17 within macrophages, leading to the shedding of receptors like MerTK and creating a form of remote inhibition characterized by a “cardiomyocyte secretome → receptor ectodomain shedding → impaired clearance” axis (27).

The integrity of MerTK is also directly linked to the active resolution of inflammation. Its activation is a prerequisite for macrophage synthesis of specialized pro-resolving mediators (SPMs), such as lipoxins and resolvins. Following MerTK cleavage, SPM generation is restricted, causing the inflammation resolution program to “stall,” which directly impacts cardiac repair outcomes (67).

Critically, this molecular-level functional inactivation is not occult. Clinical studies confirm that plasma levels of soluble MerTK (sMer), generated via cleavage, are significantly elevated in patients with acute myocardial infarction. Moreover, the degree of elevation inversely correlates with the myocardial salvage index assessed by cardiac magnetic resonance imaging (26). This indicates that sMer not only reflects the destruction of the MerTK receptor and compromised efferocytic function *in vivo* but may also serve as a circulating biomarker for the early identification of high-risk patients trapped in the vicious cycle of ‘efferocytic failure – sustained inflammation.’

It is important to note, however, that sMer remains an investigational biomarker. Currently, it is still in the preclinical and early clinical translational research phase and has not yet been incorporated into routine clinical guidelines for cardiovascular diseases. Unlike established biomarkers such as cardiac troponins (which reflect cardiomyocyte necrosis) or BNP/NT-proBNP (which indicate ventricular wall stress), sMer offers a distinct advantage: it specifically reflects the functional status of macrophages and the dynamics of the immune microenvironment. As a “functional” and “mechanistic” biomarker, sMer may provide earlier insights into the success or failure of the repair program, rather than merely quantifying the extent of myocardial injury. Thus, it holds promise for patient risk stratification and dynamic monitoring of therapeutic efficacy, rather than for acute diagnosis.

#### 4.1.2 Dysfunction in synergistic recognition and degradation modules

Efficient efferocytosis requires the precise coordination of multiple molecular machineries. The MerTK–MFG-E8 axis constitutes a prototypical synergistic recognition hub, operating in parallel with the MerTK–Gas6/PS pathway to significantly enhance the stability of the phagocytic synapse and the efficiency of internalization. The synergistic activation of both pathways maximizes the downstream PI3K–Akt signaling output, robustly driving VEGF secretion, thereby promoting angiogenesis and the formation of organized scar tissue. Defects in either pathway can lead to “decoupling of clearance from vascular repair,” resulting in ineffective repair (68).

The endpoint of the clearance process is intracellular degradation. The discovery of Legumain (Lgmn) underscores the critical importance of the lysosomal degradation pathway. Lgmn is an asparaginyl endopeptidase specifically expressed by cardiac tissue-resident macrophages. Studies demonstrate that Lgmn deficiency leads to significantly worsened cardiac function post-MI in mice, accompanied by the accumulation of apoptotic cardiomyocytes (8, 22). The underlying mechanism involves a defect in intracellular calcium mobilization within resident macrophages upon Lgmn loss, resulting in reduced cytosolic calcium. When these calcium-deficient macrophages subsequently engulf apoptotic cardiomyocytes, vesicular trafficking is impeded, and the formation of LC3-II-dependent phagolysosomes is inhibited. Consequently, the continuous, efficient cycle of phagocytic degradation of dead cardiomyocytes is interrupted (22).

Confirming its therapeutic potential, a recent interventional study demonstrated that *in situ* engineering of chimeric antigen receptor macrophages (CAR-MΦ) via targeted lipid nanoparticle delivery of Lgmn mRNA significantly enhanced their phagolysosomal cargo degradation capacity. This strategy alleviated the overload state of the efferocytic process post-MI and restored the anti-fibrotic function of macrophages, providing proof-of-concept for therapeutic strategies targeting the degradation phase (8).

#### 4.1.3 Aberrant upregulation of “don’t-eat-me” signals

Under homeostatic conditions, the CD47–SIRPα axis functions as a “don’t-eat-me” signal, protecting healthy cells. However, in the pathological setting of MI, dying cardiomyocytes aberrantly upregulate CD47 expression. Binding of CD47 to SIRPα on the macrophage surface recruits and activates tyrosine phosphatases such as SHP-1/SHP-2, which dismantles the actin network, directly inhibiting phagocytic cup closure and internalization. This creates a state of “successful recognition but failed internalization” (69).

Epigenetic research further reveals that in chronic inflammatory environments, the long non-coding RNA MIAT can act as a molecular sponge, sequestering miR-149-5p. This relieves the inhibitory effect of miR-149-5p on CD47 mRNA, leading to abnormally high CD47 protein levels. This provides a novel explanation for the hyperactivity of “don’t-eat-me” signaling post-MI (70).

Consequently, the aberrantly high expression of CD47 on dying cardiomyocytes acts like an ‘invisibility cloak,’ shielding them from clearance. Clinical prospective studies offer direct evidence for this: a prospective study involving NSTEMI patients found that elevated serum CD47 levels upon admission served as an independent risk factor for predicting major adverse cardiovascular events (MACE) both during hospitalization and in the short term post-discharge (71). This suggests that monitoring circulating CD47 levels could be a potential strategy for early warning of an impending collapse in the efferocytotic program due to overly potent “don’t-eat-me” signaling.

Nevertheless, similar to sMer, CD47 is not yet a clinically validated biomarker. Its measurement remains within the realm of translational research, with ongoing studies exploring its predictive value in larger, more diverse patient cohorts. Compared to conventional inflammatory markers like C-reactive protein (CRP), which reflects systemic inflammation, circulating CD47 provides a more localized and mechanistic readout—specifically indicating the status of the “don’t-eat-me” signaling axis within the injured myocardium. This mechanistic specificity positions CD47 as a potential tool for monitoring the efficacy of therapies targeting the CD47-SIRPα pathway and for stratifying patients who might benefit from such interventions, rather than for routine diagnostic use.

### 4.2 Interference from the inflammatory microenvironment and secondary vicious cycles

Downstream of the direct failure of molecular regulatory axes, the resultant toxic inflammatory microenvironment itself constitutes a powerful external force that suppresses efferocytosis. This leads to a functional lock within the effector cells, creating a formidable secondary vicious cycle.

#### 4.2.1 Cytokine storm and exogenous inhibition

The post-MI “cytokine storm” exerts broad-spectrum inhibitory effects on the efferocytic system. High levels of pro-inflammatory cytokines such as TNF-α and IFN-γ can directly downregulate phagocytic receptors like MerTK and AXL on the macrophage surface, fundamentally impairing their recognition capacity (56). Endogenous danger signals generated by tissue injury also play an accomplice role. For instance, low-molecular-weight hyaluronan (HA) fragments accumulating around the necrotic core act via receptors like CD44 and TLR2/4. They not only amplify the release of pro-inflammatory cytokines but also directly interfere with actin dynamics, inhibiting the phagocytic capacity of macrophages. This results in a dual assault of “pro-inflammatory amplification + direct clearance inhibition” (46).

#### 4.2.2 Failed metabolic-epigenetic reprogramming and functional lock

Efferocytosis is an energy-intensive process contingent upon subsequent cellular reprogramming. Successful efferocytosis normally triggers efficient fatty acid oxidation (FAO), elevating NAD^+^ levels and activating SIRT1. This, in turn, promotes the expression of reparative genes like IL-10, thereby stabilizing the reparative phenotype (51). Concurrently, the IL-10–STAT3–Galectin-3 axis serves as another crucial signaling pathway driving reparative phenotypic polarization (72).

However, within the post-MI milieu characterized by hypoxia, oxidative stress, and a pro-inflammatory environment, these pivotal metabolic and signaling circuits are disrupted. Consequently, macrophages become trapped in a vicious cycle of “metabolic paralysis → epigenetic lock → failure to establish a reparative phenotype.” This state of functional lock perpetuates low phagocytic efficiency while solidifying a pro-inflammatory bias. Unable to transition from a “scavenger” to a “repairer,” macrophages in this locked state become a critical node amplifying the initial injury.

### 4.3 Cascading consequences of dysregulation: from cellular debris to heart failure

The multi-dimensional dysregulation at the molecular, cellular, and microenvironmental levels described above collectively triggers an escalating and largely irreversible malignant pathological cycle. The inception of this cycle lies in the comprehensive failure of the efferocytic machinery—characterized by MerTK cleavage, aberrant CD47 signaling, and Lgmn-mediated degradation blockade—which prevents the timely clearance of massive numbers of apoptotic cardiomyocytes. This leads to their secondary necrosis and the consequent release of a flood of damage-associated molecular patterns (DAMPs), such as HMGB1 and ATP, into the interstitium (21, 22, 26).

The ensuing core pathological event is the complete loss of inflammatory control and the congenital absence of repair instructions: the released DAMPs are perpetually engaged by pattern recognition receptors, causing overactivation of the innate immune system and a dysregulated amplification of pro-inflammatory cytokines. This prevents the acute inflammation from resolving as programmed. Concurrently, failed efferocytosis severs the critical trigger for initiating tissue repair. Macrophages are deprived of the positive instructions normally conveyed via the MerTK and other pathways, resulting in a severe deficiency in the secretion of key reparative factors like IL-10, TGF-β, and VEGF. Consequently, the reparative program fails at its very inception (33, 68, 72).

This directly precipitates catastrophic structural outcomes: persistent cell death and imbalanced extracellular matrix metabolism cause the necrotic core to expand continuously. The resultant reparative scar is both weak and structurally disorganized, with significantly compromised mechanical strength, drastically increasing the risk of cardiac rupture in the acute phase. In the chronic phase, infarct expansion, diffuse fibrosis in the non-infarcted myocardium, and progressive ventricular dilation become intertwined, collectively constituting irreversible adverse cardiac remodeling (22, 26).

Ultimately, the damage at all levels converges, leading to a unified terminal outcome: the progressive deterioration of cardiac pump function. Patients inevitably advance to symptomatic end-stage heart failure, with mortality rates rising significantly (2, 22).

In summary, dysregulation of efferocytosis post-MI originates from core molecular events such as MerTK cleavage, CD47 upregulation, and impaired Lgmn-mediated degradation. This results in apoptotic cell accumulation and secondary necrosis. The DAMPs released thereby not only drive uncontrolled inflammatory amplification but, more critically, sever the essential metabolic and transcriptional reprogramming signals required to initiate tissue repair. The consequence is a state where inflammation fails to resolve, the repair program cannot launch, the necrotic core expands, adverse ventricular remodeling becomes irreversible, and the heart progresses towards failure. Furthermore, the persistently pro-inflammatory and pro-fibrotic microenvironment, in turn, further inhibits the efferocytic capacity of macrophages, thereby establishing a self-reinforcing vicious cycle of ‘efferocytic failure – sustained inflammation – failed repair’, locking the heart onto a path of pathological remodeling. Breaking this cycle is the key to effective intervention.

### 4.4 Context-dependent dual roles of key molecules: a call for balanced targeting

The preceding sections have unequivocally established the detrimental consequences of efferocytic dysfunction in the post-MI heart. However, a nuanced perspective demands recognition that the key molecules orchestrating this process are not monolithic “good” or “bad” actors; rather, their functional outcomes are profoundly context-dependent (73). A balanced therapeutic strategy must therefore aim not at maximal activation or inhibition of any single pathway, but at restoring its physiological spatiotemporal equilibrium.

CD47: Beyond a “Don’t-Eat-Me” Signal. While aberrantly upregulated CD47 on dying cardiomyocytes contributes to pathological immune evasion and impaired clearance, CD47 also fulfills essential physiological functions (69). It is constitutively expressed on healthy cells, including erythrocytes and platelets, where it serves as a marker of “self” to prevent unwarranted phagocytosis (71). More critically, CD47 plays a well-established role in platelet homeostasis, mediating interactions with thrombospondin-1 and regulating platelet activation and aggregation. Systemic, non-selective CD47 blockade, therefore, carries an inherent risk of hematological toxicities, including anemia and thrombocytopenia, as observed in oncology clinical trials (74). This duality underscores the imperative for cardiac-specific delivery systems that achieve localized therapeutic efficacy without compromising systemic CD47 function.

MerTK: A Double-Edged Sword in Chronic Inflammation. Within the acute post-MI setting, MerTK activation is unequivocally reparative, driving efferocytosis and orchestrating inflammation resolution (21, 26). However, the role of MerTK is not universally beneficial across all pathological contexts. In certain chronic inflammatory conditions and tumor microenvironments, sustained MerTK signaling on macrophages has been implicated in promoting a fibrotic phenotype and contributing to immune suppression (75, 76). For instance, in bleomycin-induced models of systemic sclerosis, macrophages with sustained MerTK activation accumulate in fibrotic tissues and directly promote fibroblast activation and collagen production (75). This context-dependent duality suggests that while enhancing MerTK function acutely post-MI is desirable, chronic, non-specific activation could inadvertently foster adverse long-term remodeling or off-target effects.

Reparative Macrophages: The Yin and Yang of Healing. The paradigm of M1 (pro-inflammatory) and M2 (reparative) macrophage polarization, while conceptually useful, oversimplifies a complex spectrum of activation states. It is increasingly recognized that the sustained or excessive presence of “reparative” macrophages can itself become pathological. In the later stages of cardiac remodeling, an overabundance of M2-like macrophages and their persistent secretion of TGF-β can drive excessive extracellular matrix deposition, leading to unfavorable fibrosis, myocardial stiffening, and diastolic dysfunction (16, 38). This illustrates the critical principle that the “dose” and “duration” of a reparative signal are as important as its presence. The goal is not to indefinitely maximize M2 polarization, but to orchestrate a transient, tightly regulated wave of repair that resolves once tissue integrity is restored.

Collectively, these examples illuminate a central tenet for the rational design of efferocytosis-targeted therapies: the objective is not to simply maximize the activity of a single pro-repair pathway, but to restore its physiological spatiotemporal dynamics (73, 74). An effective therapeutic intervention must navigate the narrow therapeutic window where efficacy is achieved without tipping the balance towards the latent pathological potential of these pleiotropic molecules. This necessitates a shift from a mindset of wholesale activation or inhibition towards one of precise modulation—fine-tuning the intensity, duration, and location of the intervention to re-establish the homeostatic equilibrium that governs successful tissue repair.

## 5 Therapeutic strategies targeting efferocytosis and future perspectives

Building upon a profound understanding of the molecular mechanisms of efferocytosis and a precise dissection of its dysregulated steps following myocardial infarction, researchers have developed multi-dimensional interventional strategies spanning molecular, cellular, and microenvironmental levels. These strategies collectively constitute a comprehensive therapeutic framework aimed at restoring the inflammation-repair balance and promoting cardiac repair. This chapter will systematically elaborate on these strategies, provide a side-by-side comparison in **Table 1**, and conclude with a prospective analysis of the field’s challenges and future directions.

**Table 1:** Comparative analysis of therapeutic strategies targeting efferocytosis.

Strategy category	Representative technologies	Unique advantages	Major risks & translational barriers	Mitigation strategies	Ideal application scenarios (time window & spatial zone)	Current stage
1. Disabling “Don’t-Eat-Me” Signal Brake	Anti-CD47 antibodies/nanobodies; Bionic nanodegraders; Engineered macrophages	Most direct mechanism; Rapid onset; Oncology experience available	Hematological toxicities (anemia, thrombocytopenia); Low cardiac targeting efficiency; Immunogenicity of protein inhibitors	Cardiac-homing peptide conjugation; Magnetic targeting; Humanization/PEGylation; Localized delivery via engineered cells	Acute phase (days 0–3), targeting infarct border zone where apoptotic cells first accumulate	Oncology: clinical trials; Cardiovascular: preclinical
2. Enhancing “Eat-Me” Signals & Clearance Capacity	PPARγ agonists; Lgmn mRNA-LNP; CAR-MΦ engineering	Targets specific efferocytosis steps; Addresses “engulfment without digestion” bottleneck	Immunogenicity of mRNA/LNP; Liver accumulation rather than cardiac targeting; Off-target Lgmn activity in non-infarcted regions	Nucleoside-modified mRNA; Cardiac-targeted LNP; Microenvironment-responsive (pH/ROS) delivery	Transition phase (days 3–7), targeting efferocytic overload specifically in infarct border zone	Advanced preclinical (Lgmn mRNA); Traditional drug mechanistic studies
3. Systematically Reprogramming Immune Microenvironment	TREM2/SIRT1 modulators; Engineered EVs delivering non-coding RNAs	Targets upstream signaling hubs; Breaks “functional lock”; Sustained effects	Complex regulatory networks; High off-target risk; Rapid EV clearance by liver/spleen; Systemic metabolic perturbation	Heart-specific metabolic checkpoint identification; CD47-mediated EV “stealth” modification; Localized delivery	Reparative phase (beyond day 7), stabilizing reparative phenotype across all zones, particularly preventing fibrosis in border zone and limiting inflammation in remote zone	Early mechanistic exploration; Some preclinical proof-of-concept
4. Intelligent Carriers & Combination Therapies	Microenvironment-responsive nanoparticles; CD47-modified “stealth” EVs; Biomimetic co-delivery systems	Enhances spatiotemporal precision; Enables “1 + 1>2” synergistic effects	Complex manufacturing; High cost; Quality control challenges; Unclear drug-drug interactions	Modular carrier platforms; AI-assisted design; Multi-stage release systems responsive to sequential cues	Personalized comprehensive therapy enabling zone-specific multi-target intervention (anti-necrotic in core, pro-efferocytic in border, homeostatic in remote)	Emerging preclinical; Rapid technology development

### 5.1 Disabling the “don’t-eat-me” signal brake

Targeting the CD47-SIRPα axis represents one of the most direct therapeutic strategies to restore the phagocytic function of macrophages (77). However, systemic blockade of this pathway may lead to hematological toxicities such as anemia and thrombocytopenia. Consequently, the development of nanotechnological and cellular engineering strategies capable of spatiotemporally precise intervention has become a research priority (78).

Among these, genetically engineered macrophages, serving as “living cell factories,” show unique promise. For instance, Tan et al. constructed engineered macrophages via lentiviral transduction. These cells not only stably overexpressed phagocytic receptors like MerTK but also produced and released anti-CD47 nanobodies *in situ*. This approach creates a localized microenvironment within the infarcted myocardium that simultaneously “lifts inhibition” and “enhances clearance,” achieving a form of “self-supplying” precision therapy (77).

In the realm of bionic nanotechnology, novel delivery systems offer a new paradigm for acute-phase intervention. Gao et al. designed a bionic nanodegrader that mimics cell membrane components and carries a small-molecule CD47 inhibitor. Critically, this system is designed to target the infarct border zone during the peak inflammatory phase (days 1-3), leveraging acid-responsive or enzyme-cleavable linkers to achieve rapid local release and degradation of CD47, thereby precisely enhancing efferocytosis at the site of greatest apoptotic cell accumulation (78). A similar precision delivery concept is exemplified by Pan et al., who developed nanoparticles modified with a cardiac-homing peptide and featuring dual responsiveness to reactive oxygen species and pH. These particles can target the delivery of an SHP1 inhibitor to the infarct zone, promote efferocytosis and reduce inflammation by downregulating downstream signaling of the CD47-SIRPα axis, ultimately improving cardiac function (79). Furthermore, Zheng et al. constructed a bionic nanorobot integrating a nanomotor with extracellular vesicles. It delivers SHP-1 siRNA to block the “don’t-eat-me” signal while utilizing the vesicles themselves to induce macrophage polarization towards a pro-efferocytic phenotype, synergistically activating efferocytic function within atherosclerotic plaques (80).

Notably, the application of CD47 blockade strategies is expanding beyond acute myocardial infarction. For example, in atherosclerosis, upregulated CD47 expression within plaques inhibits efferocytosis, leading to necrotic core expansion. Studies indicate that simultaneously blocking CD47 and the key lipid metabolism target ANGPTL3 can synergistically regulate lipid metabolism, enhance efferocytosis, and reduce lipid peroxidation, offering a new therapeutic direction for atherosclerosis (81). Furthermore, innovative approaches like macrophage-biomimetic nanoparticles co-delivering checkpoint inhibitors (e.g., SIRPα-mimicking components) and cholesterol efflux promoters (e.g., retinoic acid) have been developed. These strategies achieve a dual enhancement of efferocytosis and lipid clearance, presenting a novel and synergistic anti-inflammatory paradigm for managing atherosclerotic plaques (82).

### 5.2 Enhancing “eat-me” signals and clearance capacity

Building upon the removal of inhibitory signals, proactively enhancing the “positive” signaling of the phagocytic system is crucial for improving repair quality. This strategy encompasses interventions across the entire pathway, from bridging to degradation.

In terms of augmenting bridging molecules, research extends beyond novel protein therapies to include new discoveries concerning traditional drugs. Zhang et al. demonstrated that the traditional Chinese medicine Xin Shu Bao Tablet enhances efferocytosis and improves ventricular remodeling by upregulating the expression of the bridging molecule MFGE8 via activation of the PPARγ/MFGE8 pathway (83). This finding provides a fresh perspective for the modern study of traditional medicines.

At the lysosomal degradation stage, Legumain (Lgmn) stands out as a key target. Breakthrough work by the research team at Shandong University demonstrated that *in situ* engineering of chimeric antigen receptor macrophages (CAR-MΦ) via lipid nanoparticle (LNP)-delivered Lgmn mRNA significantly enhances their phagolysosomal cargo degradation capacity (8, 15, 22). This strategy is ideally suited for the transition phase (days 3-7), precisely when efferocytic overload and functional exhaustion peak in the border zone. By enhancing lysosomal degradation capacity, it alleviates the “blockage” in the efferocytic process and restores the anti-fibrotic function of macrophages specifically within the infarct border, where apoptotic cell burden is highest (8). This synthetic biology strategy provides robust proof-of-concept for therapies targeting the degradation phase. The approach has been patented and is actively advancing towards clinical translational research.

Furthermore, epigenetic regulation reveals its unique potential. Ciullo et al. discovered that a non-coding RNA, yREX3, within human extracellular vesicles can regulate macrophages via a novel gene methylation mechanism independent of traditional receptor-ligand interactions (84). This opens a new avenue for developing nucleic acid-based drugs to modulate efferocytosis.

### 5.3 Systematically reprogramming the immune microenvironment

The highest-level strategy focuses on the systemic remodeling of the post-infarction immune microenvironment, aiming to create a milieu that continually promotes repair through the coordinated regulation of multiple cell types and signaling pathways.

Intelligent cell and vesicle therapies represent a major branch of this approach. For instance, apoptotic cell-derived nanovesicles, acting as intrinsic “eat-me” signals, can preemptively “educate” macrophages and have been shown to effectively prevent adverse cardiac remodeling (85). Innovatively, Wei et al. developed CD47-modified extracellular vesicles, which temporarily “deceive” the mononuclear phagocyte system, significantly prolonging the half-life of therapeutic vesicles (86). This “stealth” technology offers a generalizable solution to the challenge of rapid *in vivo* clearance of nanomedicines.

In the realm of metabolic reprogramming and combination therapy, research reveals deeper regulatory networks. Gong et al. discovered that TREM2 promotes cardiac repair by enhancing macrophage efferocytosis and modulating Slc25a53 and carbohydrate metabolism (87), establishing a direct link between efferocytosis and a specific mitochondrial metabolic axis. This approach is particularly suited for medium-to-long-term management, targeting the reparative phase (beyond day 7) to stabilize the reparative macrophage phenotype and prevent the transition to chronic inflammation and adverse remodeling across all myocardial zones. Meanwhile, multi-target synergistic strategies demonstrate significant advantages. Song et al. developed a biomimetic nanocarrier capable of simultaneously targeting ferroptosis and efferocytosis, achieving a synergistic therapeutic effect by reducing “bad death” while enhancing “good clearance” (88). Furthermore, N2 neutrophils have been confirmed to reprogram macrophages towards a pro-healing phenotype (40). This underscores that the efficacy of efferocytosis depends not only on the macrophages themselves but is also intricately linked to the collaborative regulation by other immune cells within the microenvironment.

### 5.4 Critical analysis of therapeutic strategies

Although the aforementioned strategies hold significant promise, an objective critical analysis is essential to advance their clinical translation. **Table 1** provides a systematic comparison and evaluation of these strategies, revealing several common challenges facing the field. The primary paradoxical challenge lies in the trade-off between specificity and safety. The most direct-acting strategies (e.g., systemic CD47 blockade) are often accompanied by significant off-target side effects, while safer approaches (e.g., metabolic reprogramming) face serious questions regarding their specificity and interventional efficacy.

Beyond this fundamental dilemma, three specific translational hurdles warrant deeper scrutiny. First, immunogenicity represents a major concern for biologic-based approaches. mRNA/LNP platforms, engineered cell therapies, and exogenous proteins (e.g., SIRPα-Fc fusion proteins) can provoke host immune responses, potentially neutralizing therapeutic efficacy and causing adverse reactions. Mitigation strategies include humanization of protein sequences, PEGylation to reduce immunogenicity, and the use of autologous cell sources for cell-based therapies. Second, delivery efficiency to the infarcted heart remains a formidable obstacle. Nanoparticles face multiple barriers: low cardiac targeting efficiency after systemic administration, rapid clearance by the mononuclear phagocyte system, and impaired vascular perfusion within the infarct core due to microvascular obstruction. Emerging strategies to overcome these barriers encompass physical targeting (e.g., magnetic guidance), biomimetic cloaking (e.g., cell membrane-coated nanoparticles), and cascade-release systems engineered to respond to specific cues within the infarct microenvironment. Third, off-target effects extend beyond the well-recognized hematological toxicities of CD47 blockade. Metabolic reprogramming agents may inadvertently perturb systemic energy homeostasis, while localized Lgmn overexpression in non-infarcted regions could disrupt normal tissue homeostasis or promote unintended proteolytic activities. These considerations underscore the need for rigorous preclinical evaluation of tissue-specific effects.

Secondly, many cutting-edge solutions (e.g., *in situ* mRNA engineering, cell therapies) are highly dependent on complex technological platforms. Their high production costs, stringent quality control standards, and currently unclear regulatory pathways collectively constitute a formidable barrier to clinical translation. Finally, the predictive power of preclinical models has inherent limitations. The widely used young, healthy animal models fail to adequately simulate the complex pathophysiological environment of clinical patients, who are typically elderly and present with multiple comorbidities. This discrepancy can lead to an overestimation of therapeutic benefits and an underestimation of potential toxicities in preclinical studies. Therefore, identifying these common issues is a crucial prerequisite for guiding the next generation of therapeutic strategies toward clinical success.

In summary, these challenges collectively point to a central dilemma: how to achieve the optimal balance between ‘precision’ (the right cell, at the right time, in the right location, with the right intensity) and ‘translational viability’ (safety, cost-effectiveness, and accessibility) within the complex and dynamic pathological environment of myocardial infarction.

### 5.5 Challenges and future directions

Despite the immense therapeutic potential of targeting efferocytosis, its clinical translation faces systematic scientific and technological hurdles, necessitating comprehensive innovation from conceptual frameworks to practical tools. Future breakthroughs will depend fundamentally on enhancing temporal precision in intervention. This entails developing intelligent delivery systems capable of dynamically sensing changes in the infarct microenvironment (e.g., pH, reactive oxygen species, specific enzyme activity) and mounting an adaptive response. Such systems could precisely augment efferocytosis during the early inflammatory phase to promote resolution, while avoiding pathological fibrosis resulting from its overactivation in the later reparative stages.

Concurrently, achieving breakthroughs in cell-targeting specificity is another critical bottleneck. This requires the deep integration of precision navigation tools, such as cardiac-homing peptides and cell-specific promoters, to enable the spatially precise modulation of specific immune cell subsets within the lesioned area. In terms of clinical translation pathways, innovative exploration is imperative. Establishing a biomarker evaluation system for efferocytic function, incorporating markers like sMerTK and CD47, would provide objective metrics for patient risk stratification and treatment efficacy monitoring. Furthermore, a deeper investigation into the synergistic effects between efferocytosis modulation and traditional cardiovascular therapeutic strategies may yield “1 + 1>2” therapeutic outcomes.

Looking ahead, combination therapy is poised to become the predominant paradigm. The organic integration of efferocytosis-targeting strategies with neuroendocrine inhibitors, specific anti-inflammatory agents, or other cell death-targeting approaches holds promise for multi-nodal, synergistic blockade of the vicious pathological cycle post-MI. Simultaneously, next-generation therapies based on genetically engineered cells, intelligent nanocarriers, and exosomes/microvesicles are propelling the field from concept to clinic. The convergence of these advances with cutting-edge analytical technologies, such as single-cell multi-omics and spatial transcriptomics, will empower us to construct high-resolution, dynamic maps of the post-MI immune microenvironment. This, ultimately, will usher in the era of personalized medicine.

By coherently integrating these forward-looking directions, we can realistically aspire to transform efferocytosis from a core hub of inflammation resolution into an effective therapeutic target for functional cardiac repair, thereby opening a novel therapeutic landscape for improving outcomes in patients with myocardial infarction.

## 6 Summary and perspectives

This review systematically argues for the central “directorial” role of efferocytosis in post-myocardial infarction repair. It transcends a mere “scavenger” function, orchestrating the sequential evolution of the immune microenvironment from an inflammatory storm to reparative reconstruction through precise spatiotemporal regulation. From the initial “find-me/eat-me” signaling cascade, through the synergistic internalization mediated by TAM-TIM receptor collaboration during the execution phase, to the decisive metabolic-transcriptional reprogramming, efferocytosis meticulously choreographs the entire cardiac repair program. Establishing this pivotal hub role not only clarifies the internal logic determining the success or failure of post-MI repair but also provides critical spatiotemporal targets for interventional strategies.

Current research frontiers are dedicated to translating our profound decoding of this “directorial program” into actionable “directorial skill” for precise intervention. This involves not only identifying key “actors” (cells and molecules) but also mastering their “cue” (temporal sequence) and “stage positioning” (spatial localization). It is noteworthy that the pathophysiological significance of efferocytosis extends far beyond myocardial infarction. In atherosclerosis, its dysfunction directly contributes to necrotic core formation within plaques and disease progression (10, 16). In oncology, the CD47-mediated “don’t eat me” signal is a key mechanism of immune evasion (89). In autoimmune diseases, defective efferocytosis is closely linked to autoantigen exposure and the breakdown of immune tolerance (90). This conservation across diverse disease entities suggests we are engaging with a fundamental immunoregulatory program essential for maintaining organismal homeostasis.

However, compared to these chronic or systemic pathologies, efferocytosis post-myocardial infarction faces a uniquely brutal “stress test.” It unfolds within a terminally differentiated organ with minimal regenerative capacity and must contend with an intense, acute wave of cell death within an extremely compressed timeframe. This uniqueness means that even a slight dysfunction in efferocytosis can rapidly precipitate the self-reinforcing vicious cycle of “efferocytic failure – sustained inflammation – failed repair” (detailed in Chapter IV), thereby imposing an almost exacting demand for spatiotemporal precision in its regulation.

Looking forward, the in-depth exploration of the efferocytic “directorial program” is catalyzing a fundamental paradigm shift in cardiovascular therapeutic philosophy: moving from the traditional, passive suppression of injury signals towards an active, programmed guidance of the repair process. Realizing this paradigm evolution hinges on breakthroughs in three key areas. First, the development of intelligent delivery systems capable of dynamically sensing and responding to changes in the infarct microenvironment. This would enable the precise deployment of “directorial instructions” (e.g., CD47 inhibitors, *Lgmn* mRNA) within the correct therapeutic windows (e.g., the transition phase). Second, utilizing cardiac/cell-specific targeting tools to ensure spatial precision of interventions, thereby minimizing systemic toxicity. Third, establishing a patient stratification and efficacy monitoring system based on circulating biomarkers like sMerTK and CD47, which would provide a basis for crafting individualized “therapeutic scripts.”

A rational approach to spatial targeting necessitates a “three-zone differentiated therapy” concept. In the necrotic core, where efferocytosis is fundamentally compromised by the extreme microenvironment, strategies should prioritize anti-inflammatory and anti-necrotic interventions to limit damage. In the infarct border zone—the primary battlefield for efferocytosis—therapeutic efforts must focus on enhancing efferocytic clearance and promoting angiogenesis, precisely at the time of peak apoptotic burden. In the remote, non-infarcted myocardium, the goal is to maintain homeostatic surveillance, preventing the spread of inflammation and the activation of maladaptive fibrosis. This spatially nuanced strategy recognizes that a single therapeutic approach cannot address the distinct pathological demands of each zone; rather, a coordinated, multi-pronged intervention tailored to the unique microenvironment of each region is required.

Despite facing formidable technical challenges in areas such as cell targeting and temporal control, this philosophical evolution—from a static “nodal target” mindset to a dynamic “programmatic re-editing” approach—undoubtedly represents a progressive direction in therapeutics. Ultimately, through the deep integration of multidisciplinary technologies to decode and finely tune this intrinsic repair program, we hold the promise of pioneering a novel therapeutic paradigm aimed not just at myocardial infarction, but at a range of inflammatory and degenerative diseases. This paradigm would seek to rebuild tissue homeostasis rather than merely delay damage. It challenges us to transcend our role as mere “observers” of the microscopic world and strive to become skilled “conductors” of the exquisite symphony that governs tissue fate—where efferocytosis serves as the principal conductor, ensuring every cellular section plays in harmony and in time.
